# Oxidation of Arsenite by Epoxy Group on Reduced Graphene Oxide/Metal Oxide Composite Materials

**DOI:** 10.1002/advs.202001928

**Published:** 2020-09-23

**Authors:** Qiantao Shi, Li Yan, Chuanyong Jing

**Affiliations:** ^1^ State Key Laboratory of Environmental Chemistry and Ecotoxicology Research Center for Eco‐Environmental Sciences Chinese Academy of Sciences Beijing 100085 China; ^2^ School of Environmental Science and Engineering Shandong University Qingdao 266237 China

**Keywords:** adsorption, arsenic, graphene, oxidation

## Abstract

Reduced graphene oxide/metal oxide (rGO/MO) hybrid has been widely used as a catalyst, while dissolved oxygen or radicals are generally recognized as the oxidant. This study finds that the adsorbed arsenite (As(III)) on rGO/MO is oxidized to arsenate (As(V)) in the absence of other oxidants or radicals. The oxidation of As(III) is observed on varying rGO/MOs, including rGO/MOs composited of different types of reduced graphene oxide (rGO) and metal oxide. The epoxy group on rGO acts as the oxidant, evidenced by the significant correlation between the consumption of epoxy group and oxidation of As(III). Meanwhile, metal oxide provides adsorption sites for As(III) during the adsorption–oxidation process. Based on a combination of spectroscopic measurements and computational calculation, a possible pathway for As(III) oxidation by rGO/MO is proposed: the oxygen atom in the epoxy group is bonded to the adsorbed As^III^O_3_, which is consequently oxidized to As^V^O_4_. Overall, this study proves the role of rGO/MO as an oxidant, which opens a new perspective on future studies using rGO/MO as a catalyst for the oxidation reaction.

## Graphical abstract

Oxidation of adsorbed arsenite (As(III)) on reduced graphene oxide/metal oxide (rGO/MO) occurs in the absence of other oxidants or radicals. Reduced graphene oxide (rGO) acts as an oxidant rather than a catalyst for As(III) oxidation, while metal oxide provides sites for As(III) adsorption. During the adsorption–oxidation process, the epoxy group on rGO binds to As^III^O_3_ and forms As^V^O_4_.

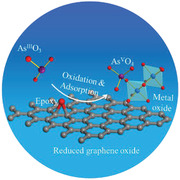

## 1 Introduction

Reduced graphene oxide/metal oxide (rGO/MO) hybrid has attracted increasing attention,^[^ ^1^ ^]^ and been widely applied in the fields of energy,^[^ ^2^ ^]^ catalysis,^[^ ^3^ ^]^ and environment.^[^ ^2^, ^4^ ^]^ Most of the existing studies on rGO/MO focused on addressing the importance of its functional oxygen groups, which are introduced in the graphene oxide (GO) production process, and cannot be totally removed during the reduction from GO to reduced graphene oxide (rGO).^[^ ^3^, ^5^ ^]^ Current studies usually consider these residual oxygen groups on rGO/MO as adsorptive or catalytic sites whereas the molecular oxygen^[^ ^6^ ^]^ or other oxidants^[^ ^7^ ^]^ are recognized as the electron acceptor. However, it is reported that GO can be an oxidant, for example, the use of GO as a chemical oxidant in polymers fabrication.^[^ ^8^ ^]^ Moreover, it is reported that the epoxy group could break from GO and form O_2_,^[^ ^9^ ^]^ which consequently acts as an oxidant. Theoretical calculation by the density functional theory (DFT) also indicates that the epoxy group could react with C—H and change to hydroxyl group.^[^ ^10^ ^]^

Although these studies showed the rationale of epoxy group as an oxidant, no experimental study has demonstrated the oxidant role of epoxy group on rGO/MO, due to the lack of direct evidence for the consumption of epoxy group in the oxidation process.^[^ ^3a^ ^]^ The consumption of epoxy group is difficult to be monitored or characterized because of the low residual epoxy content in rGO/MO. Moreover, the general concern of most studies is the change of reactants and radicals rather than rGO/MO itself because of the conventional understanding of rGO/MO as a catalyst.

The objective of this study was to explore the function of the epoxy group on rGO/MO as an oxidant. In this study, we first found the oxidation of arsenite (As(III)) by a composite material of rGO/lanthanum hydroxide (rGO/LO) without the presence of light and molecular oxygen. To further clarify the oxidant role of rGO, different types of rGO/MO, including varying rGOs and metal oxides, were synthesized and tested for the As(III) oxidation. The correlation between the epoxy group and As(III) oxidation by these rGO/MOs was illustrated by monitoring the real‐time consumption of epoxy group and oxidation of As(III) using in situ flow cell attenuated total reflectance Fourier transform infrared spectroscopy (ATR‐FTIR). Moreover, the quantitative correlation analysis between the amount of epoxy group and the oxidation ability of rGO/MOs was performed by X‐ray photoelectron spectroscopy (XPS) and X‐ray absorption near edge structure (XANES) techniques. Finally, a possible adsorption‐oxidation pathway is proposed based on the spectroscopic evidence and further confirmed by the DFT calculation. The discovery of epoxy group on rGO/MO as an oxidant opens a new chapter in our understanding of the role of epoxy group, which is helpful for the future studies regarding the application of rGO/MO on the oxidative degradation or adsorption of environmental pollutants.

## 2 Results and Discussions

### 2.1 Observation of As(III) Oxidation by rGO/LO

**Figure 1:**
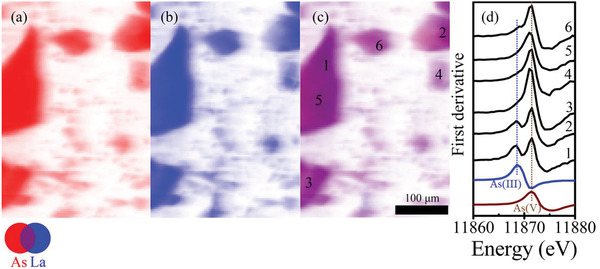
Micro‐X‐ray fluorescence (µ‐XRF) mapping of a) As, b) La, and c) As+La distribution on rGO/LOa after adsorption of As(III). The numbers in (c) indicate the positions for µ‐XANES characterization, which are also shown in (d); XANES spectra of As(III) (blue line) and As(V) (wine line) standards are also shown in panel (d); the vertical dash lines in blue and wine represent the As(III) and As(V) feature, respectively.

The oxidation of As(III) was first observed in a micro‐X‐ray absorption (µ‐XAS) spectroscopy characterization for As(III) adsorption on one type of rGO/LO, namely rGO/LOa. The rGO/LOa was composed of rGO and nanorod‐like La(OH)_3_, which was fabricated by a simultaneous formation of La(OH)_3_ and reduction of GO to rGO. The synthesis procedure was detailed in Figure S1, Supporting Information, and the basic physicochemical properties of rGO/LOa were described in Text S1 and S2, Supporting Information, and shown in Figures S2–S4, Supporting Information. The distribution of As and La on rGO/LOa with adsorbed As(III) was analyzed by micro‐X‐ray flThuorescence (µ‐XRF) mapping (**Figure** **1**a,b), while the chemical valence of arsenic (As) in interested sites was identified by micro‐X‐ray absorption near edge structure (µ‐XANES, Figure 1d) analysis.

Interestingly, the adsorbed As(III) was found to be oxidized to As(V) after adsorption onto rGO/LOa, evidenced by the observed As(V) feature in µ‐XANES spectra (indicated by the vertical wine dashed line in Figure 1d). Moreover, different extents of As(III) oxidation were observed on those randomly selected sites (sites 1 to 6 shown in Figure 1c). Specifically, the As(III) µ‐XANES feature at 11 869 eV (indicated by the vertical blue dashed line in Figure 1d) is obvious on sites 1 and 2, but inconspicuous on sites 3, 4, 5, and 6, implying different As(V)/As(III) ratios between sites 1–2 and 3–6 (Figure 1d). However, the La content at sites 1–2 and sites 3–6 has no significant difference (Figure 1b). Therefore, the µ‐XRF and µ‐XANES results indicate two important points: 1) As(III) was oxidized to As(V) after adsorption on rGO/LOa; 2) La(OH)_3_ had an insignificant effect on As(III) oxidation.

Please note, the distribution of As is consistent with that of La, as evidenced by the overlapped As and La elemental distribution (Figure 1c) and the correlation analysis (*R* ^2^ = 0.968, Figure S5, Supporting Information). These observances indicate As is mainly adsorbed on La(OH)_3_. Overall, the µ‐XRF and µ‐XANES results prove that La(OH)_3_ provides adsorption sites but is not responsible for the oxidation of As(III).

### 2.2 Responsible Oxidant for As(III) Oxidation

The oxidation of As(III) might result from radicals produced in the catalytic process by rGO/MO. To evaluate if the radicals could possibly contribute to the oxidation of As(III) in this study, electron paramagnetic resonance (EPR) was employed to test the existence of superoxide radical (O_2_·^−^) and hydroxyl radical (·OH) in rGO/LOa solutions. The obtained results (Text S3, Figure S6, Supporting Information) indicated that no radical was produced in the solutions, which excludes the possibility that the radical is responsible for the oxidation of As(III). Therefore, it is anticipated that the oxidant could be rGO/LOa in the system. It should be noted that all the adsorption experiments were conducted in the absence of O_2_ or light.

To verify the hypothesis of the oxidant role of rGO, other rGO/MO materials were synthesized by varying the type of metal (hydr)oxide, namely rGO/alumina (rGO/AO, Figure S1, Supporting Information) and rGO/titanium dioxide (rGO/TO, Figure S1, Supporting Information). The three rGO/MO materials, that is, rGO/AO, rGO/TO, and rGO/LOa, were then characterized by XANES at a low temperature (<70 K) after the adsorption of As(III). The obtained first derivative XANES spectra (Figure S7, Supporting Information) show the As(V) feature, indicating that all three rGO/MOs are capable to oxidize As(III). Therefore, the oxidation ability should be attributed to the common component of these rGO/MO materials, which is rGO. Please note, the As(V) feature on µ‐XANES spectra is more significant than that on bulk XANES spectra, due to the focused beam and exposure to air at room temperature for µ‐XANES.

On the other hand, to illustrate how the change in rGO might affect the oxidation of As(III), other rGO/LOs materials were synthesized by changing the synthetic method of rGO while that of La(OH)_3_ was consistent. The difference between these rGO/LO materials lies in the different contents of the functional oxygen groups resulting from varying synthetic methods, which is discussed later. In this way, the relationship between the functional oxygen groups and the oxidation ability of rGO/LO can be further analyzed. These rGO/LO materials including rGO/LOa, rGO/LOb, rGO/LOc, and rGO/LOd were also characterized by XANES after As(III) adsorption (Figure S7, Supporting Information). Interestingly, As(V) peaks with different intensities appeared on these spectra, implying varying degrees of As(III) oxidation by the different rGO/LOs, which confirmed that the functional oxygen groups are responsible for the oxidation of As(III).

**Figure 2:**
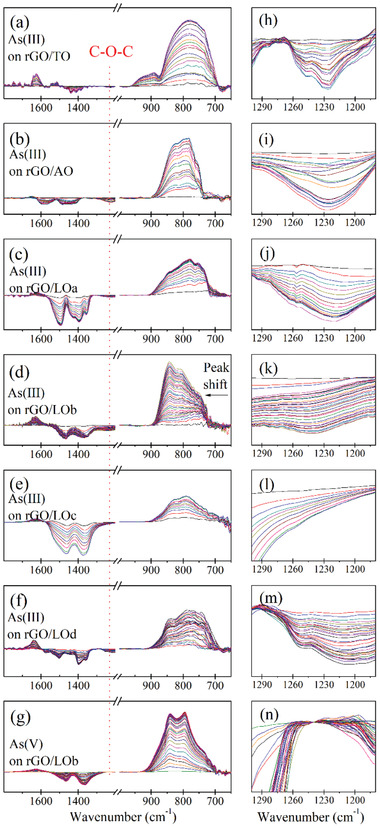
In situ ATR‐FTIR spectra of As(III) adsorption on a) rGO/TO, b) rGO/AO, c) rGO/LOa, d) rGO/LOb, e) rGO/LOc, and f) rGO/LOd, and g) As(V) adsorption on rGO/LOb; h–n) red dashed line indicates the IR band of epoxy group, which is further magnified in the figures on right; the significant As‐O band shift is marked in panel (d).

To further demonstrate which functional oxygen group relates to the As(III) oxidation, As(III) adsorption on rGO/MOs was monitored using the in‐situ flow cell ATR‐FTIR technique. As shown in **Figure** **2**, the IR peaks at 680–900 cm^−1^ increase over time, resulting from the adsorption of As(III) onto rGO/MO. Generally, the IR band at 680–830 cm^−1^ mainly results from the As(III)‐O vibration, while the band at 730–900 cm^−1^ is due to the As(V)‐O vibration.^[^ ^11^ ^]^ Here, the appearance of IR band at 830–900 cm^−1^ confirmed the oxidation of As(III), which is in agreement with the XANES results. Moreover, the kinetics of the As‐O band increasing have been analyzed (Text S4 and Figure S8, Supporting Information). The reaction kinetic of the As‐O band of As(III) on rGO/MOs follows a zero order chemical reaction (*R* ^2^ = 0.961–0.996) better than typical adsorption kinetics of pseudo first order (*R* ^2^ = 0.839–0.961) or pseudo second order (*R* ^2^ = 0.186–0.957) models. The fitted rate constants range from 2.2–6.2 × 10^−5^ abs·min^−1^. As a control test, the IR increasing of As(V) adsorption on rGO/LOb follows pseudo first order (*R* ^2^ = 0.956) rather than zero order (*R* ^2^ = 0.897). This difference suggests that the appearence of As‐O bands for As(III) on rGO/MO might result from adsorption–oxidation rather than adsorption only. In addition, a significant As‐O band shift from 740 to 840 cm^−1^ was observed during the As(III) adsorption on rGO/LOb. This band shift results from the more significant oxidation of As(III), because rGO/LOb showed a stronger oxidation ability than other rGO/MOs, which was evidenced by the higher intensity of the As(V) feature in XANES patterns for rGO/LOb (Figure S7, Supporting Information).

The negative peaks at 1300–1600 cm^−1^ result from the decreasing hydroxyl group content on metal oxide, due to the widely known ligand exchange mechanism, that is, the exchange between the hydroxyl group on metal oxide and anions in solution. Meanwhile, a negative peak at 1220–1260 cm^−1^ appeared on the IR spectra for As(III) adsorption (Figure 2a–f). This band corresponds to the C‐O‐C stretch of epoxy group,^[^ ^5^, ^12^ ^]^ indicating the consumption of epoxy group during the As(III) adsorption process. As a control, the As(V) adsorption on rGO/LOb shows no negative peak around 1220–1260 cm^−1^ (Figure 2g).

The correlation between the consumption of epoxy group and oxidation of As(III) was further confirmed by a 2D correlation spectroscopy (COS) analysis. In a 2D‐COS synchronous map, the appearance of a cross‐peak indicates the correlation between two FTIR peaks at two different wavenumbers.^[^ ^13^ ^]^ In this case, a cross‐peak with wavenumber of 1220–1260 cm^−1^ (IR band of epoxy group) and 840 cm^−1^ (IR band of As(V)) is observed (Figure S9, Supporting Information). The negative cross‐peak indicates a significant correlation between the decreasing epoxy and increasing As(V), due to the consumption of epoxy group and the oxidation of As(III), respectively. Therefore, the 2D‐COS analysis overall evidenced that the epoxy group was consumed and responsible for the oxidation of As(III) on rGO/LOb.

### 2.3 Quantitative Correlation between Epoxy Group and As(III) Oxidation

The quantitative correlation between the amount of epoxy group and oxidation ability of rGO/MO was further analyzed. First, the amount of epoxy group on rGO/LOa, rGO/LOb, rGO/LOc, and rGO/LOd materials was measured by X‐ray photoelectron spectroscopy (XPS). The results show that rGO/LOb exhibits higher epoxy content (34.5%) than other rGO/LO materials (9.9–13.1%) (Figure S10, Table S2, Supporting Information). In addition, due to the high oxidation ability of rGO/LOb, different types of rGO/LOb were synthesized by varying the amount of NaBH_4_, and they were named as rGO/LOb, rGO/LOb‐2, rGO/LOb‐3, rGO/LOb‐4, and rGO/LOb‐5. All these rGO/LOb materials still contain more epoxy groups (24.3–37.3%) than other rGO/LO materials (i.e., rGO/LOa, rGO/LOc, and rGO/LOd). These results demonstrate that the oxidation ability of rGO/LO is mainly determined by the different reductants used in the synthetic procedure: NaBH_4_ prefers to reduce O=C—OH and C—OH rather than C—O—C, whereas N_2_H_4_ favors the reduction towards C—O—C.^[^ ^5a^ ^]^ Then, the oxidation ability was evaluated by calculating the amount of As(V) on these rGO/LO materials after adsorption of As(III). Specifically, the amount of As(V) was obtained via multiplying the total amount of adsorbed As acquired from the batch experimental results by the percentage of As(V) obtained from the XANES linear combination fitting analysis (Figure S11, Table S3, Supporting Information).

**Figure 3:**
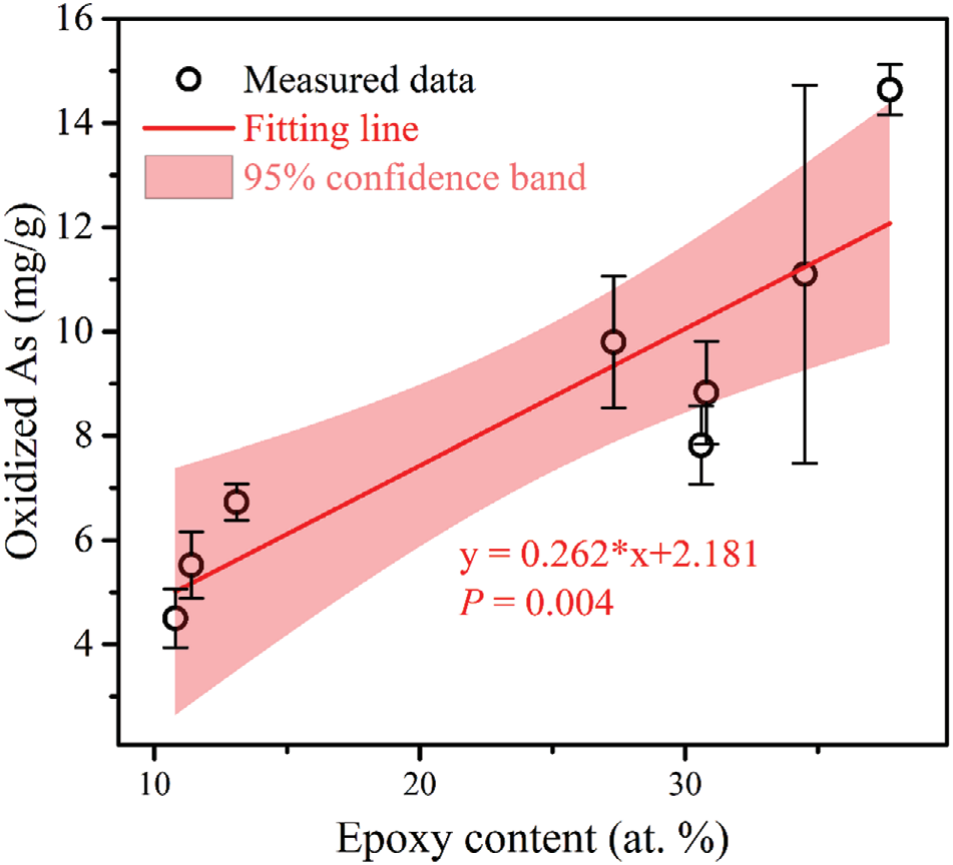
Quantitative correlation between the amount of As(V) and epoxy group for As(III) adsorption on eight types of rGO/LO materials (i.e., rGO/LOa, rGO/LOb, rGO/LOc, rGO/LOd, rGO/LOb‐2, rGO/LOb‐3, rGO/LOb‐4, and rGO/LOb‐5); the oxidized As content is the average of duplicate samples (with a total sample size of 16), which is detailed in Table S3, Supporting Information; the error bar represents the standard error of the duplicate samples; *p* < 0.05 was considered as a significant correlation.

As shown in **Figure** **3**, a significant linear correlation (*P* = 0.004) was obtained between the amount of As(V) and epoxy group on the eight rGO/LO materials, which provides affirmative evidence that the epoxy group is the oxidant in the adsorption–oxidation process for As(III) on rGO/LO.

### 2.4 Mechanisms of Adsorption–Oxidation for As(III) by rGO/LO

**Figure 4:**
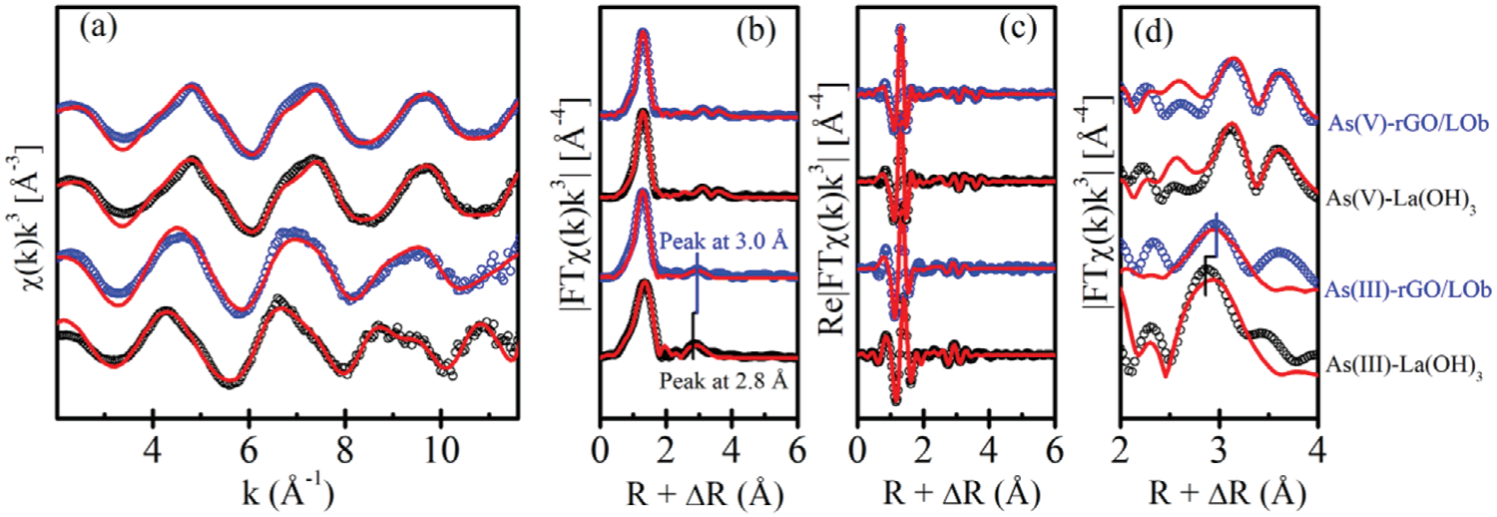
a) Normalized *k* ^3^‐weighted experimental (circles) and simulated (lines) As K‐edge EXAFS spectra of As(III) and As(V) adsorption on La(OH)_3_ and rGO/LOb, b) the corresponding Fourier transformed magnitude, and c) real parts of Fourier transform; d) highlights of the FT magnitude fitting shown in part b at the R range from 2 to 4 Å; vertical lines in (b) and (d) highlight the difference of the second FT peak for As(III) adsorption on La(OH)_3_ and rGO/LOb.

To reveal the mechanism of As(III) adsorption–oxidation on rGO/LO materials, the molecular structures of As(III) and As(V) adsorption on rGO/LO were investigated by the extended X‐ray absorption fine structure (EXAFS, **Figure** **4**). Samples of As(III) and As(V) adsorption on La(OH)_3_ were also analyzed as controls. The rGO/LOb was selected as a representative rGO/LO material due to its high oxidation ability. The EXAFS fitting results reveal the monodentate mononuclear and bidentate binuclear configurations for As(V) and As(III) adsorption on La(OH)_3,_ respectively (Table S4, Supporting Information). In addition, a third shell of La at 3.86 Å was observed for As(V) adsorption on La(OH)_3_, which is due to the surface precipitation of As(V) with La^3+^.^[^ ^14^ ^]^ The complexation structure for As(V) adsorption on rGO/LOb showed the same configuration as that on La(OH)_3_, indicating that the addition of rGO did not influence the adsorption structure.

However, the As(III) adsorption on rGO/LOb is different from that on La(OH)_3_. Specifically, the second Fourier transformed (FT) peak of As(III) adsorption on rGO/LOb (uncorrected radial distance: ≈3.0 Å, Figure 4d) is longer than that of As(III) adsorption on La(OH)_3_ (uncorrected radial distance: ≈2.8Å, Figure 4d). This difference results from the oxidation of As(III) after its adsorption on rGO/LOb, and the adsorption structure changes from bidentate binuclear to monodentate mononuclear configuration. To perform the EXAFS fitting for As(III) on rGO/LOb, the As(III) oxidation is taken into consideration, which means 85% of As(III) and 15% of As(V) (based on XANES results, Table S3, Supporting Information) is employed in the fitting. The high quality of the EXAFS fitting (R‐factor = 0.018, Table S4, Supporting Information) indicates that the As(III) and oxidized As(III) still bond with La(OH)_3_ rather than rGO.

Based on the experimental results and spectroscopic evidence, we proposed the adsorption‐oxidation process for As(III) on rGO/LO surface: As(III) in the solution was first adsorbed by La(OH)_3_, and then oxidized by the neighboring epoxy group on rGO.

**Figure 5:**
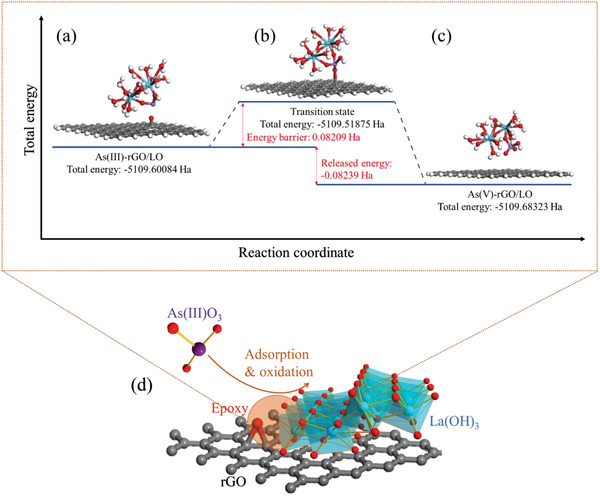
Proposed mechanisms for the adsorption–oxidation of As(III) by epoxy group on rGO/LO based on the DFT calculation: a) Calculated adsorption structures for As(III) and c) As(V) adsorption on rGO/LO, and b) the transition state obtained by transition state search calculation; part d emphasizes that the adsorption–oxidation occurs in the hybrid interface between rGO and La(OH)_3_, where is marked as wine color; the epoxy group, La(OH)_3_, As^III^O_3_, and rGO are indicated by the labels shown in part d. The interatomic distance between the O atom in epoxy group and As atom is ≈2.96 Å in part a, which is shortened to 1.65 Å in part c. The energy barrier was calculated by the equation of *E*_barrier_ = *E*_transition state_ (total energy shown in (b))‐*E*_reactant_ (total energy shown in (a)), and the released energy was calculated by the equation of *E*_released_ = *E*_product_ (total energy shown in (c)) − *E*_reactant_ (total energy shown in (a)).

To verify the proposed mechanism, the DFT calculation for As(III) adsorption‐oxidation on rGO/LO was studied using DMol^3^ (**Figure** **5**). The 26‐atom hydrogen‐terminated graphene sheet includes 96 carbon atoms, and one oxygen atom is used to simulate the epoxy group on rGO. The La(OH)_3_ cluster is built up with an edge‐sharing cluster, which is similar to our previous DFT study for As(III) and As(V) adsorption on LaOOH.^[^ ^15^ ^]^ The oxygen coordination number of 9 for La is derived from the XRD result.^[^ ^16^ ^]^ The adsorption configurations obtained from the EXAFS fitting are used to build up the adsorbed As(III) and As(V). Moreover, for As(V)‐rGO/LO complexes, the O atom in the epoxy group on rGO bonds to the As atom, representing the oxidation from As(III)(AsO_3_) to As(V)(AsO_4_). The calculated total energy of As(V)‐rGO/LO complex (−5109.683 Ha, Figure 5) is lower than that of As(III)‐rGO/LO (−5109.601 Ha), suggesting a more stable adsorption structure for As(V) than As(III). Please note, an energetic value higher than 0.01 Ha is considered as significant in this study, because the error of the DFT calculation is usually in 20 kJ mol^−1^ (corresponding to 0.0076 Ha).^[^ ^17^ ^]^ Moreover, the transitional state (TS) search calculation for the As(III) oxidation showed that the energy barrier for oxidizing As(III) to As(V) on rGO/LO is 0.08209 Ha. This value is slightly lower than the released energy from the whole adsorption–oxidation process (0.08239 Ha). Hence, the released energy from the whole procedure can oxidize As(III), suggesting that the adsorption–oxidation of As(III) to As(V) on rGO/LO is an energetically spontaneous reaction.

Overall, the reaction of the adsorption–oxidation of As(III) by rGO/MOs can be briefly summarized by the following steps: i) As(III)O_3_ was adsorbed onto rGO/MOs by complexing with metal oxides (in the case of La(OH)_3_: Figure 5a); ii) in the hybrid interfaces between rGO and La(OH)_3_, the adsorbed As(III)O_3_ was bonded to epoxy group on rGO, forming As(V)O_4_ (Figure 5b); iii) the newly formed As(V) was detached from rGO but still adsorbed on metal oxide surfaces (Figure 5c). Please note, considering that a short distance (i.e., within ≈3 Å, Figure 5a) is essential for the bonding between adsorbed As(III) on metal oxides and the epoxy group on rGO, it is reasonable to hypothesize that this oxidation occurs in the hybrid interfaces between metal oxides and rGO (Figure 5d). Therefore, only 7.3–23.6% of As(III) (Table S3, Supporting Information) was oxidized to As(V), and the distribution of As species is not uniform on rGO/LO (Figure 1).

## 3 Conclusion

This work illustrates that the epoxy group on rGO/MO can oxidize As(III) during the adsorption process, implying a novel mechanism for the oxidation reaction in the presence of rGO/MO. The direct evidence is provided by a significantly quantitative correlation between the consumption of epoxy group and oxidation of As(III), as well as a theoretical calculation of the energy change in the adsorption–oxidation process. During the past decade, rGO/MO materials have been used to catalyze oxidization reactions in numerous studies (e.g., oxidative degradation of toxic organic compounds),^[^ ^18^ ^]^ but little attention has been paid to its possible role as an oxidant. Therefore, the information gained from this study could guide researchers through re‐thinking the oxidation mechanism when using rGO/MO as a catalyst. Moreover, considering that the rGO/MO materials have also been widely used as adsorbents for pollutants removal (e.g., heavy metals adsorption),^[^ ^19^ ^]^ this study offers new insights into the adsorption of pollutants by using rGO/MO, in which the possible degradation resulting from oxidation might occur.

## 4 Experimental Section

##### Synthesis of Graphene Oxides

The graphene oxide (GO) used in this study was synthesized using the Hummers method^[^ ^20^ ^]^ and another method reported by Daniela et al.,^[^ ^12a^ ^]^ which is shown in Figure S1, Supporting Information, and detailed in Supporting information.

##### Synthesis of Different Types of rGO/MO

In this study, to explore the oxidant role of rGO, various types of rGO/MO were synthesized, including different rGOs and different metal oxides. The overall procedures are shown in Figure S1, Supporting Information. Specifically, three different rGO/MOs were synthesized and named as rGO/LO, rGO/AO, and rGO/TO. The letters of “LO,” “AO,” and “TO” represent the lanthanum hydroxide, alumina, and titanium dioxide, respectively. Moreover, different reductants were used to synthesize rGO/LO, resulting in different types of rGO and the same type of La(OH)_3_, namely, rGO/LOa, rGO/LOb, rGO/LOc, and rGO/LOd. The synthetic procedures are also detailed in the Supporting Information (shown as Figure S1, Supporting Information).

##### Characterizations of XRD, SEM, TEM, Raman Scattering, ESR, and XPS

The obtained rGO (synthesized using the Daniela method), rGO/LOa, rGO/AO, and rGO/TO were characterized by X‐ray powder diffraction (XRD, X'Pert PRO MPD, PANalytical, The Netherlands), which was recorded at 40 kV, 100 mA using a Cu‐target tube (1.5418 Å) and a graphite monochromator. Scans were made in the 2*θ* range of 5° to 90° with a step size of 0.01° and a count time of 2 s per step. Analysis of the XRD patterns was performed using the PDF‐2 reference database from the International Center for Diffraction Data (ICDD) database.

The morphology of the rGO (synthesized using the Daniela method), rGO/LOa, and La(OH)_3_ was characterized using a field emission scanning electron microscope (SU 8020, Hitachi, Japan), and a high resolution transmission electron microscope (JEM2010, JEOL, Japan). These three materials were further characterized using a Raman spectrometer (Enwave Optronics, Inc., United States) with a 4 cm^−1^ resolution at 532 nm excitation energy.

The electron spin resonance (ESR) signal of the radicals spin trapped by 5,5‐dimethyl‐1‐pyrroline‐*N*‐oxide in the solution of 5 g L^−1^ rGO/LOa was recorded on a JEOL JES FA200 Xband spectrometer (Tokyo, Japan) under photoirradiation with a 500 W mercury lamp (Ushio, USH 500D). The settings were the center field at 323.3 mT, microwave frequency at 9054.6 MHz, and power at 0.998 mW.

The XPS (ESCALAB 250, Thermo Fisher Sci., United States) was used to investigate the content of functional groups of all rGO/LO materials, including rGO/LOa, rGO/LOb, rGO/LOb‐2, rGO/LOb‐3, rGO/LOb‐4, rGO/LOb‐5, rGO/LOc, and rGO/LOd. XPS results were analyzed by XPS data processing and peak fitting was performed using the XPSPeak4.1 software package.

##### µ‐XRF and µ‐XANES Analyses

To explore the distribution of La and As, as well as the oxidation of As(III) on rGO/LOa, the rGO/LOa material with adsorbed As(III) was analyzed using synchrotron‐based µ‐XRF and µ‐XANES techniques at beamline 15U at Shanghai Synchrotron Radiation Facility (SSRF), China. The sample was prepared in batch adsorption experiments with 100 mg L^−1^ As(III) in the presence of 2 g L^−1^ rGO/LOa in 0.01 mol L^−1^ NaCl solution at pH 7. The adsorption experiments were conducted with N_2_ gas purging and without the presence of light. After 24 h mixing, the solids were separated by filtration, freeze‐dried under vacuum, and deposited between two layers of Kapton tape. The monochromator was set at 14 keV to collect µ‐XRF maps. The beam size was 3.5 × 3.5 µm^2^, the dwell time per pixel was 1 s, and the step size was 3.5 µm. The peak intensities for As and La were collected at each pixel of µ‐XRF maps with the designated area. As K‐edge (11 867 eV) µ‐XANES spectra were collected from the spots of interest in the µ‐XRF map.

##### In Situ Flow Cell ATR‐FTIR

To investigate the change of the functional groups on rGO/MO during the As(III) adsorption process, the in situ flow cell attenuated total reflectance Fourier transform infrared spectroscopy (ATR‐FTIR) measurements were performed using a Thermo‐Nicolet Nexus 6700 FTIR spectrometer equipped with a horizontal attenuated total reflectance cell (PIKE Tech) and a liquid‐nitrogen‐cooled mercury‐cadmium‐telluride detector. A multibounce ZnSe ATR crystal with 45° beveled faces (infrared angle of incidence, *θ*) was used and the infrared spectra were collected using 256 scans per spectrum at a resolution of 4 cm^−1^.

The collection and treatment of the spectra for As(III) and As(V) adsorption on rGO/MOs were similar to the procedure in the previous publications.^[^ ^15^, ^21^ ^]^ Briefly, the adsorbent film was deposited on the ZnSe crystal by applying 500 µL of the adsorbent suspension (1.5 g L^−1^) and drying at room temperature. Prior to use, the film was rinsed with DI water to remove loosely deposited particles. A 0.01 mol L^−1^ NaCl solution at pH 7 was passed through the flow cell at a rate of 0.5 mL min^−1^ until there was no further change in the IR spectra. A background spectrum was then collected that represented the absorbance of the ZnSe crystal and deposited adsorbent with the flow of 0.01 mol L^−1^ NaCl solution. Then, solutions of 50 mg L^−1^ As(III) and 20 mg L^−1^ As(V) in 0.01 mol L^−1^ NaCl were passed through the flow cell at pH 7, respectively. Spectra were recorded as a function of time until the adsorption reached equilibrium for both the solutions (at least 5 h). The solutions were purged with N_2_ without the presence of light in the flow cell.

##### 2D Correlation Spectroscopy Analysis for IR Spectra

2D COS was utilized to identify whether the consumption of epoxy group correlated to the oxidation of As(III). After essential smoothness and baseline correction using the Omnic 9.0 software (Thermo‐Nicolet Inc., United States), the 2D‐COS analysis was performed for the obtained dynamic IR spectra of As(III) adsorption on rGO/LOb in the 2Dshige software (Shigeaki Morita, KwanseiGakuin University, 2004–2005).

##### X‐Ray Absorption Spectroscopy

Samples for the X‐ray absorption spectroscopic analysis (XAS) were prepared in batch adsorption experiments with 100 mg L^−1^ As(III) in the presence of 2 g L^−1^ rGO/MO in 0.01 mol L^−1^ NaCl solution at pH 7. All rGO/MO materials (including rGO/TO, rGO/AO, rGO/LOa, rGO/LOb, rGO/LOc, rGO/LOd, rGO/LOb‐2, rGO/LOb‐3, rGO/LOb‐4, and rGO/LOb‐5) were studied, and the experiments were conducted in dark. The samples of As(III) and As(V) adsorbed on La(OH)_3_, and As(V) adsorbed on rGO/LOb were also prepared under the same condition, and used as controls. The As K‐edge (11 867 eV) spectra were collected at beamline 01C1 at the National Synchrotron Radiation Research Center (NSRRC), Taiwan. An energy range of −200–1000 eV from the K‐edge was used to acquire the spectra with a standard Lytle detector at cryogenic temperatures (<70 K) using a helium cryostat to prevent beam‐induced oxidation. Three to six scans were collected for each sample, inspected for overall quality, and averaged to improve the signal/noise ratio. The XANES spectra were further analyzed using a linear combination fit in the Athena program in the Demeter computer package. The EXAFS data were analyzed using the Athena and Artemis programs in the Demeter computer package and as detailed in Supporting Information (Text S5, Supporting Information).

##### Computational Methodology

The geometry optimization, total energy calculations, and transitional state (TS) search for As(III) adsorption–oxidation by rGO/LO were performed within the DFT framework and implemented with the DMol^3^ code.^[^ ^22^ ^]^ The structure of rGO was represented by C_96_H_26_O and the charge is set at −2. An oxygen bonded to two carbon atoms represented the epoxy group (C—O—C), and an edge‐sharing and 9 oxygen coordinated La_2_O_16_H_24_ cluster is used to represent La(OH)_3_. The Perdew–Burke–Ernzerhof exchange‐correlation function within the generalized gradient approximation was employed in the DFT calculation.^[^ ^23^ ^]^ The effective‐core‐potentials method was used to deal with relativistic effects. A double numerical basis set was used together with polarization functions (DNP). A smearing of 5 × 10^−3^ Ha to the orbital occupation was applied to achieve accurate electronic convergence. Self‐consistent‐field procedures were performed with a convergence criterion of 1 × 10^−6^ Ha on the total energy. The convergence tolerance of energy, force, and displacement in the geometry optimizations was 1 × 10^−5^ Ha, 2 × 10^−3^ Ha Å^−1^, and 5 × 10^−3^ Å, respectively. All structures were optimized without any symmetry restriction and spin‐unrestricted self‐consistent field was performed during the calculation. The transition state (TS) search based on the complete linear synchronous transit/quadratic synchronous transit method was used to find the maximum energy, and then the TS confirmation based on the nudged elastic band method was used to calculate the energy barriers.^[^ ^24^ ^]^

##### Statistical Analysis

Statistical analysis was performed to identify the correlation between the amount of epoxy group the oxidation ability of the rGO/MOs, that is, the amount of epoxy group obtained from XPS (Figure S9 and Table S2, Supporting Information) and the oxidized As(III) (e.g., As(V)) percentage derived from XANES analysis (Figure S10 and Table S3, Supporting Information). Duplicate XANES data (a total of 16 data points) were performed and the average was used for the statistical analysis, showing mean values ranging from 4.5 to 14.64 mg g^−1^ with a standard error ranged from 0.35 to 3.63 mg g^−1^. The correlation analysis was conducted by linear regression using OriginPro 2016 (OriginLab, United States). A two sided test was conducted and *p* < 0.05 was considered of statistical significance.

## Conflict of Interest

The authors declare no conflict of interest.

## Supporting information

Supporting Information
