# Supplementary material for: Oxidation of Arsenite by Epoxy Group on Reduced Graphene Oxide/Metal Oxide Composite Materials
Source: Adv Sci (Weinh). 2020 Sep 23;7(21):2001928. doi: 10.1002/advs.202001928 (PMC7610301; doi:10.1002/advs.202001928)
Supplement: Supplementary file 1 — Supporting Information [file ADVS-7-2001928-s001.pdf]

© 2020 Wiley-VCH GmbH

ADVANCED SCIENCE
Open Access

Supporting Information

for *Adv. Sci.*, DOI: 10.1002/advs.202001928

Oxidation of Arsenite by Epoxy Group on Reduced Graphene Oxide/Metal Oxide Composite Materials

*Qiantao Shi, Li Yan, and Chuanyong Jing**

Supporting Information of

Oxidation of arsenite by epoxy group on reduced graphene oxide/metal oxide composite materials

Qiantao Shi, Li Yan, Chuanyong Jing*

State Key Laboratory of Environmental Chemistry and Ecotoxicology, Research Center for Eco-Environmental Sciences, Chinese Academy of Sciences, Beijing 100085, China

*Corresponding author: E-mail: cyjing@rcees.ac.cn
Tel: +86 10 6284 9523 Fax: +86 10 6284 9523

Contents:

Figure S1 on Page S2: Synthesis of rGO/MO;
Text S1 on Page S4: Results of characterizations for rGO/LOa;
Figure S2 on Page S5: Electron microscope images;
Text S2, Figure S3, and Table S1 on Page S6: Results of Raman analysis;
Figure S4 on Page S7: Results of XRD;
Figure S5 on Page S8: Correlation analysis of μ-XRF results;
Text S3 and Figure S6 on Page S9: Results of EPR for radical tests;
Figure S7 on Page S10: First derivative of XANES analysis;
Text S4 and Figure S8 on Page S11-S13: Reaction kinetics analysis;
Figure S9 on Page S14: 2D-COS analysis of FTIR spectra;
Figure S10 on Page S15 and Table S2 on Page S16: C1s XPS analysis;
Figure S11 on Page S17: XANES LCF fitting;
Table S3 on Page S18: A summary of XANES LCF fitting and XPS fitting;
Text S5 on Page S19-20 and Table S4 on Page S21: EXAFS fitting.

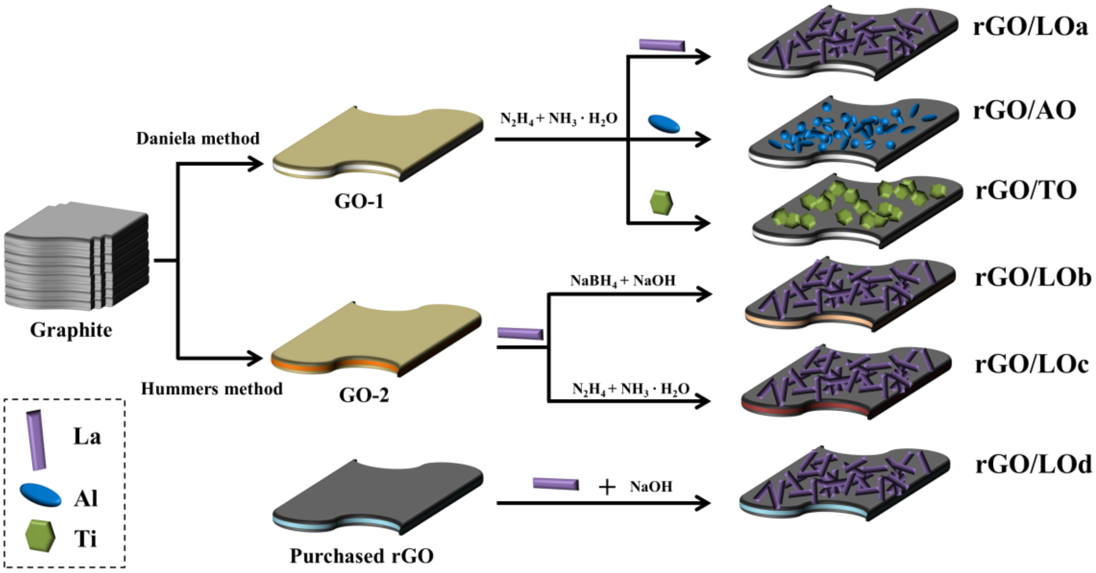

Figure S1. Scheme of procedures for synthesizing various rGO/MOs.

Synthesis of graphene oxides

Regarding the Hummers' method, 23 mL H_2SO_4 was added into 1 g graphite flake and 0.5 g $NaNO_3$ in a 250 mL three-neck flask with a stir bar. After the mixture was cooled in an ice bath for 20 min, 3 g $KMnO_4$ was slowly added and the mixture was maintained in the ice bath for another 2 h. The mixture was heated to 35°C in the water bath and kept for 1 h. Then, the synthesized viscous dispersion was slowly poured into 250 mL deionized (DI) water (resistivity > 18.2 MΩ·cm). A 30% H_2O_2 solution (10 mL) was slowly added into the mixture. The obtained mixture was then filtered using the PTFE membrane with a 0.22 μm pore size and washed in the sequence by 1 L DI water, 1 L 10% HCl, and 2 L DI water. The resulting filtered cake was freeze-dried under vacuum condition.

Regarding the Daniela method, a mixed solution of H_2SO_4 (180 mL) and H_3PO_4 (20 mL) was added slowly into 1.5 g graphite flake and 9 g $KMnO_4$ in a three-neck flask. This mixed solution was then heated to 50°C and kept mixing for 12 h. The flask was then cooled down to room temperature and the resulting mixture was poured onto 200 g ice. The synthesized mixture was then filtered, washed, and dried using the same procedures as those in the Hummers' method.

Synthesis of different types of rGO/MO

To synthesize rGO/LOa, 0.04 g GO synthesized using the Daniela method was added into 150 mL DI water in a three-neck flask. A solution of 1.75 g $La(NO_3)_3·6H_2O$ in 25 mL DI water (La-solution) was added into the GO solution. The pH was then adjusted to 10 using the ammonia solution ($NH_3·H_2O$), and 100 μL N_2H_4 was added into the solution. The solution was then heated to 90°C using a water bath. After 4 h mixing, the mixture was filtered and washed using DI water until the conductivity below 100 μS cm^{-1}. The separated solids were freeze-dried under vacuum. The same procedure was also used to synthesize other rGO/MOs, in which 0.97 g $Ti(SO_4)_2$ and 2.70 g $Al_2(SO_4)_3$ were used to synthesize the rGO/TO and rGO/AO, respectively.

For rGO/LOb and rGO/LOc, the GO fabricated by the Hummers method was used. In addition, the reductants used to synthesize rGO/LOb are $NaBH_4$ and NaOH, while those for rGO/LOc are N_2H_4 and $NH_3·H_2O$. The purchased rGO from Sinopharm (China) was used to synthesize rGO/LOd.

Furthermore, to obtain rGO/LOb with different contents of functional groups, 0.4, 0.02, 0.1, 0.2, and 0.8 g $NaBH_4$ were used to synthesize rGO/LOb, and rGO/LOb-2, rGO/LOb-3, rGO/LOb-4, and rGO/LOb-5, respectively.

All reagents were obtained from Sinopharm, China.

Text S1. Results of characterizations for rGO/LOa.

The electron microscope image (Figure S1-a) shows that the rGO films are agglomerated, agreeing well with previous studies.[1] The pristine $La(OH)_3$ is in the shape of nanowire (Figure S1-b), which changed to nanorod after being deposited on rGO (Figure S1-c), suggesting that rGO inhibited the growth of $La(OH)_3$. The Raman analysis also confirmed the crystalline change of $La(OH)_3$ after being composited with rGO (Figure S3, Table S1, and Text S2). The major crystal of rGO/LOa is $La(OH)_3$, as evidenced by the X-ray diffraction pattern shown in Figure S4. In addition, the co-existence of amorphous rGO sheet and (1 0 1) crystalline facet of $La(OH)_3$ was revealed in the HRTEM image (Figure S1-d).

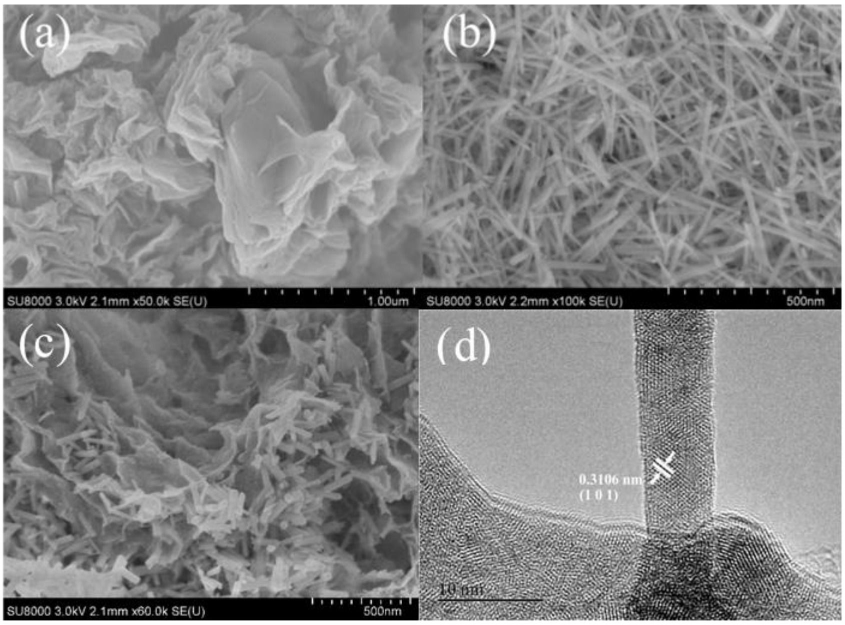

Figure S2. Scanning electron microscope (SEM) images of rGO (a), $La(OH)_3$ (b), and rGO/LOa (c); high resolution transmission electron microscope (HRTEM) image of rGO/LOa (d). The inset label in d shows the (1 0 1) crystalline facet of $La(OH)_3$ nanorods, with an interplanar distance of 0.3106 nm.

Text S2. Results of Raman analysis.

The Raman analysis was employed to further characterize the properties of these three materials (Figure S3). The peaks observed on Raman spectra were assigned and summarized in Table S1. The peaks of D-band, G-band, 2D-band, and S3 agreed well with those of the rGO reported in the previous studies.[2] The ratio of peak intensities of D- and G-band (I_D/I_G) is 1.73-1.76, indicating that GO was reduced to rGO.[2b] The peaks on Raman spectra of $La(OH)_3$ are observed at 281, 339, 452, 610, and 1071 cm^{-1}, which also agrees well with the previous reports.[3] However, it is interesting that the $La(OH)_3$ deposited on rGO shows new peaks at 396 and 855 cm^{-1}. This difference is mainly due to the change in the ratio of the crystalline faces, which suggest the chemical composite of rGO and $La(OH)_3$, as is observed from the FESEM images.

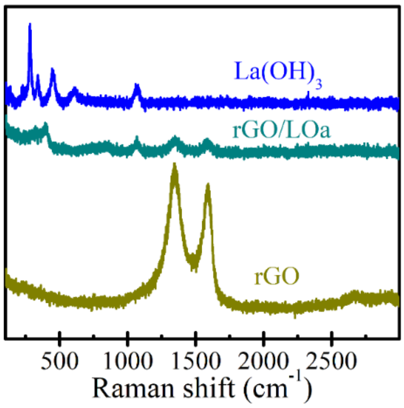

Figure S3. Raman spectra of rGO, rGO/LOa, and $La(OH)_3$.

Table S1. Summary and assignment of Raman spectra for rGO, rGO/LOa, and $La(OH)_3$.

Peak position (cm^{-1})	Assignment	rGO	rGO/LOa	$La(OH)_3$
281				√
339				√
396			√	
452				√
610				√
855			√	
1071			√	√
1344	D-band	√	√	
1594	G-band	√		
~2680	2D	√		
2920	S3	√		

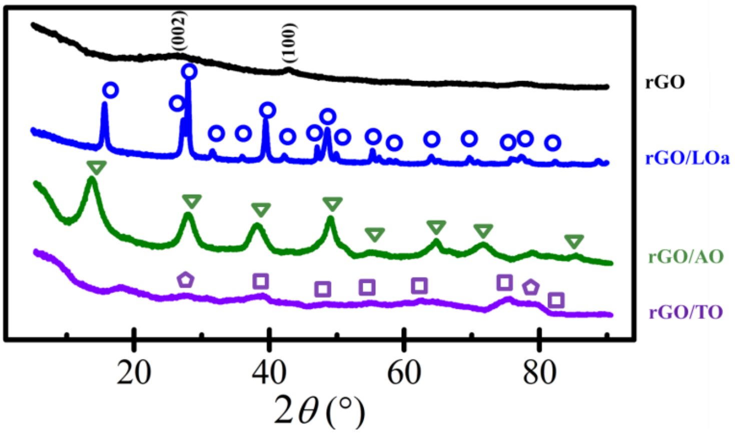

Figure S4. XRD patterns of rGO (black line), rGO/LOa (blue line), rGO/AO (green line), and rGO/TO (violet line). Labels in this figure: blue circle, $La(OH)_3$, PDF No. 36-1481; green triangle, AlO(OH), PDF No. 49-0133; violet square, anatase, PDF No. 21-1272; violet pentagon, rutile, PDF No. 21-1276; the (002) and (001) crystalline faces of rGO are also labeled in this figure.

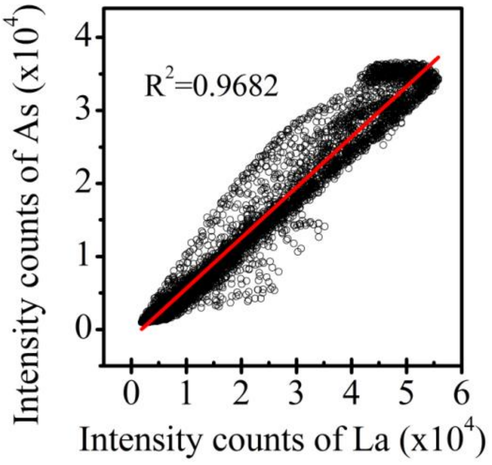

Figure S5. Correlation between As and La in μ-XRF mapping for As(III)-rGO/LOa.

Text S3. Evaluation of the presence of radicals.

The chemical 5, 5-dimethyl-1-pyrroline-N-oxide (DMPO) was used to capture the radicals with 5 g/L rGO/LOa in water under dark condition, which is the same condition used for adsorption and IR experiments. The solution containing 1 mM Co(II) solution, 100 mM H_2O_2, and 2% dimethyl sulfoxide (DMSO) was used to produce $O_2{\cdot}^-$ radical, while the solution containing 1 mM Fe(II) and 100 mM H_2O_2 was used to produce ·OH. The results showed that no radical was detected in solutions containing rGO/LOa, excluding the possibility that radical would oxidize As(III) to As(V) during the adsorption process.

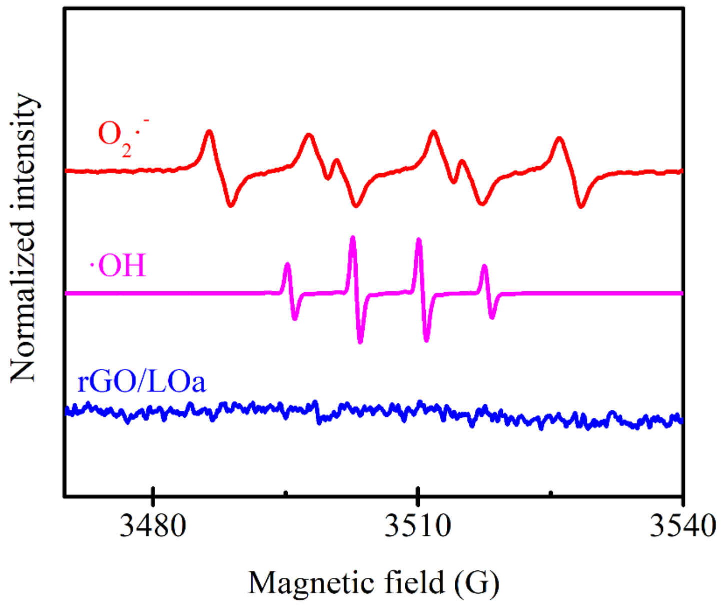

Figure S6. EPR spectra of rGO/LOa, superoxide radical ($O_2{\cdot}^-$), and hydroxyl radical (·OH).

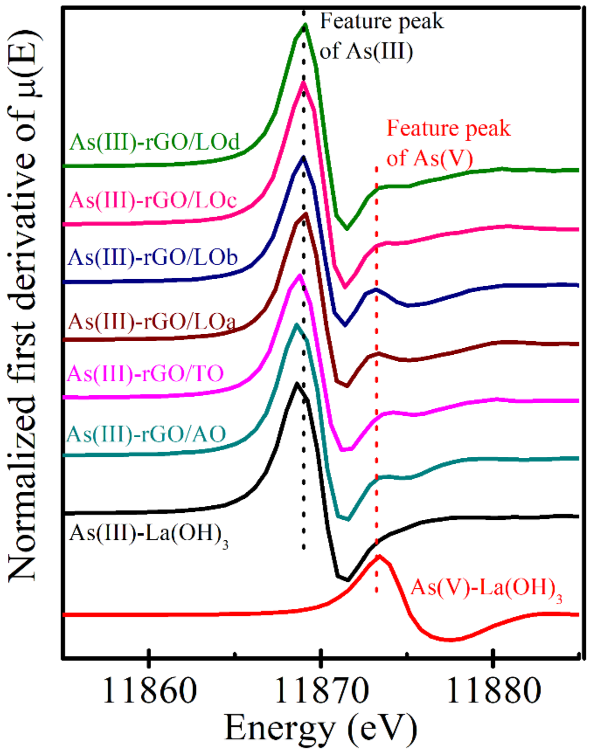

Figure S7. First derivative XANES spectra for As(III) adsorption on rGO/AO, rGO/TO, rGO/LOa, rGO/LOb, rGO/LOc, rGO/LOd, and $La(OH)_3$, as well as As(V) adsorption on $La(OH)_3$.

Text S4. The reaction kinetics of the adsorption-oxidation of As(III) on rGO/MOs were explored by analyzing the in-situ FTIR data. The increasing of IR absorbance at the highest peak of As-O band (i.e., band at 680-900 cm^{-1}) are plotted against the reaction time (Figure SX). Zero order (Eq 1), pseudo first order (Eq. 2), and pseudo second order (Eq 3) models were used to describe the kinetics for the reactions:

$$q_t = k_0 t \quad (1)$$

$$q_t = q_e(1 - e^{-k_1 t}) \quad (2)$$

$$q_t = \frac{q_e^2 k_2 t}{1+q_e k t} \quad (3)$$

where q_e and q_t are the IR absorbance of As-O band (highest peak at 680-900 cm^{-1}) at equilibrium and at any time t (min), respectively. The k_0 (abs·min^{-1}), k_1 (min^{-1}) and k_2 (abs^{-1} min^{-1}) are the rate constants for the zero order, pseudo first order, and pseudo second order models, respectively.

To fit the zero order model, the data were analyzed by linear regression using Eq 1; whereas the data were analyzed by linear regression using Eqs 4 and 5 to fit the pseudo first order and pseudo second order models, respectively.

$$\log(q_e - q_t) = \log(q_e) - \frac{k_1}{2.303} t \quad (4)$$

$$\frac{t}{q_t} = \frac{t}{q_e} + \frac{1}{q_e^2 k_2} \quad (5)$$

(Eqs 4 and 5 are the linear form of Eqs 2 and 3, respectively)

The fitting results are shown in Figure S8, as well as the fitting coefficients and rate constants. Obviously, the increasing of As-O band for As(III) on rGO/MOs follows

zero order model (R^2 = 0.961-0.996) better than pseudo first order (R^2 = 0.839~0.961) or pseudo second order (R^2 = 0.186~0.957) models. On the other hand, the increasing of As-O band of As(V) adsorption on rGO/LOb follows pseudo second order (R^2 = 0.956) and pseudo second order (R^2 = 0.929) models better than zero order (R^2 = 0.897) model. This difference further confirmed the different mechanisms of the appearance of the As-O band between As(III) and As(V) onto rGO/MOs: the As-O bands of As(III) onto rGO/MOs resulted from the adsorption-oxidation whereas the As-O bands of As(V) onto rGO/MOs resulted from the adsorption only.

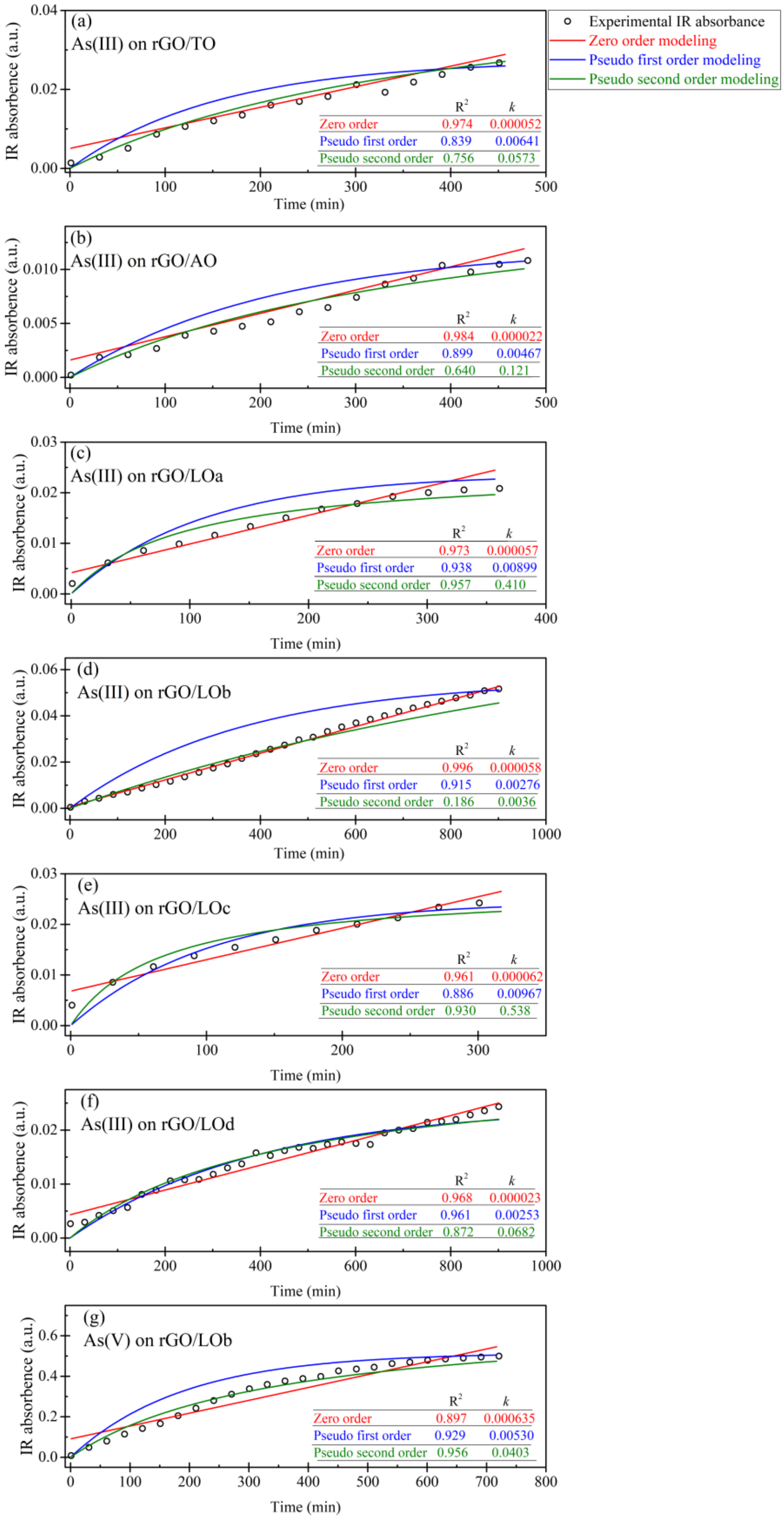

Figure S8. Reaction kinetics of As(III) adsorption-oxidation on rGO/TO (a), rGO/AO (b), rGO/LOa (c), rGO/LOb (d), rGO/LOc (e), and rGO/LOd (f), as well as As(V) adsorption on rGO/LOb (g); the absorbance data prior to equilibrium were derived from the in-situ FTIR spectra (Figure 2). Inset tables show the fitting coefficient (R^2) and the fitted rate constants.

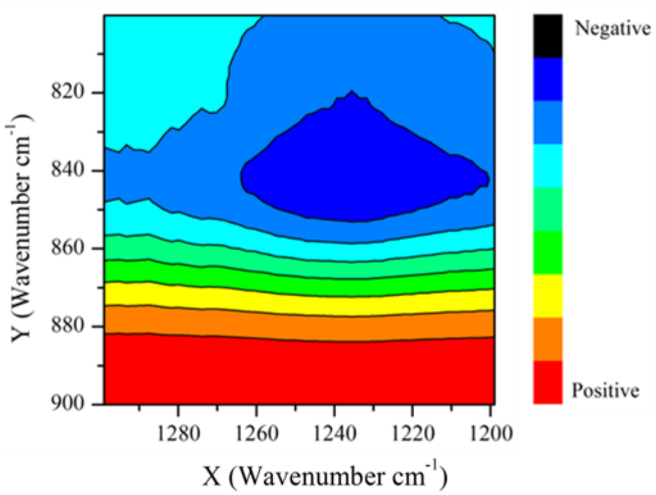

Figure S9. Synchronous 2D-COS maps for As(III) adsorption on rGO/LOb. The original FTIR spectra are shown in Figure 3-d.

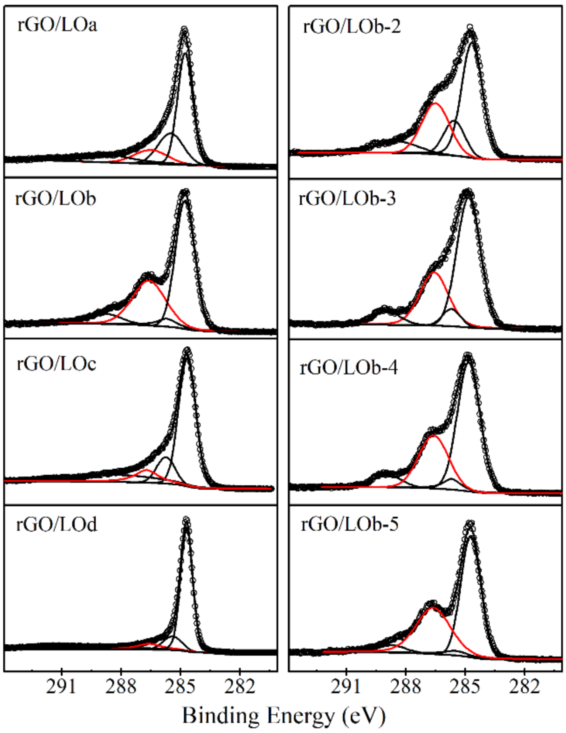

Figure S10. Peak fitting of XPS C1s spectra of eight rGO/LO materials.

Table S2. Atomic ratio of different functional groups obtained from C1s XPS spectra.

Materials	Relative atomic percentage (%) (Fitting of the C1s peak binding energy [eV])*			
	C=C (284.8)	C-OH or C-C (285.7)	C-O-C (286.8)	O=C-OH (288.9)
rGO/LOa	49.0	22.2	13.1	15.8
rGO/LOb	52.9	5.1	34.5	7.5
rGO/LOc	59.0	12.9	11.4	16.7
rGO/LOd	70.1	12.6	9.9	7.3
rGO/LOb-2	48.9	14.1	27.3	9.7
rGO/LOb-3	56.4	5.8	30.6	7.2
rGO/LOb-4	57.1	5.1	30.8	7.0
rGO/LOb-5	51.6	3.6	37.7	7.1

*The C1s XPS spectroscopy was aligned by referring to the main C=C peak at 284.8 eV. The assignments of these peaks followed the literature[4].

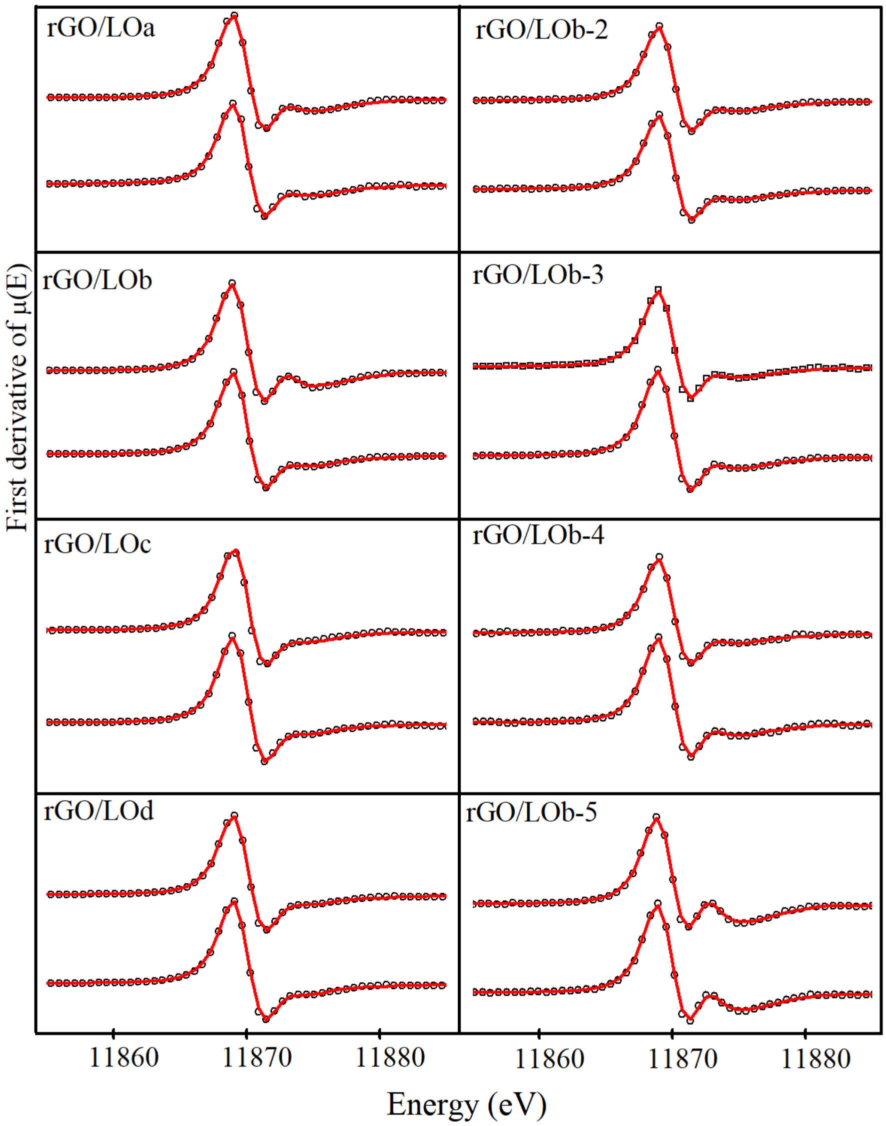

Figure S11. XANES spectra of As(III) adsorption on rGO/LOa, rGO/LOb, rGO/LOc, and rGO/LOd, as well as rGO/LOb-2, rGO/LOb-3, rGO/LOb-4, and rGO/LOb-5. Duplicate samples were analyzed for each material.

Table S3. List of adsorbed As, XANES LCF results, and epoxy group content from XPS results.

	Batch experiment	XANES fitting					XPS fitting
Samples	Total As adsorbed (mg/g)	As(V) (%)	As(III) (%)	As(V) content (mg/g)	Average As(V) content (mg/g)	Standard error	Epoxy group percentage (%)
As(III)-rGO/LOa	45.0<br>54.5	14.4<br>12.8	85.6<br>87.2	6.48<br>6.98	6.73	0.35	13.1
As(III)-rGO/LOb	75.0<br>74.0	18.2<br>11.5	81.8<br>88.5	13.7<br>8.51	11.1	3.63	34.5
As(III)-rGO/LOc	60.4<br>66.4	8.4<br>9.0	91.6<br>91.0	5.07<br>5.98	5.52	0.64	11.4
As(III)-rGO/LOd	56.3<br>62.0	7.3<br>7.9	92.7<br>92.1	4.11<br>4.90	4.50	0.56	10.8
As(III)-rGO/LOb-2	83.5<br>67.0	12.8<br>13.3	87.2<br>86.7	10.7<br>8.91	9.80	1.26	27.3
As(III)-rGO/LOb-3	60.5<br>51.0	13.8<br>14.3	86.2<br>85.7	8.35<br>7.29	7.82	0.75	30.6
As(III)-rGO/LOb-4	51.5<br>64.5	18.5<br>12.6	81.5<br>87.4	9.53<br>8.13	8.83	0.99	30.8
As(III)-rGO/LOb-5	63.0<br>63.5	22.7<br>23.6	77.3<br>76.4	14.3<br>15.0	14.64	0.48	37.7

Text S5. EXAFS data analysis

The extended X-ray absorption fine structure (EXAFS) analysis was performed using the ATHENA and ARTEMIS programs in the Demeter computer package.[5] The analytical procedure was similar to our previous studies.[6] The raw data measured in intensities were converted to μ(E), and averaged spectra were used in the analysis. The EXAFS signal $\chi(k)$ was extracted from the measured data using the AUTOBK algorithm[7] where k is the photoelectron wave number. The primary quantity for EXAFS is χ(k), which is the oscillations as a function of photoelectron wave number. χ(k) is weighted by k^3 to account for the dampening of oscillations with increasing k. The different frequencies in the oscillations in χ(k) correspond to different near neighbor coordination shells which can be described and modeled according to the EXAFS equation

$$\chi(k) = \sum_j \frac{N_j f_j(k) e^{-2k^2\sigma_j^2}}{kR_j^2} \sin[2kR_j + \delta_j(k)]$$

where f(k) and δ(k) represent the photoelectron backscattering amplitude and phase shift, respectively, N is the number of neighboring atoms, R is the distance to the neighboring atom, and σ^2 is the Debye-Waller factor representing the disorder in the neighbor distance. The k^3 weighted EXAFS spectrum in k-space ($Å^{-1}$) is Fourier transformed (FT) in R-space (Å). The experimental spectra were fitted with single-scattering theoretical phase-shift and amplitude functions calculated with the *ab initio* computer code FEFF6[8] using atomic clusters generated from the crystal structures of $LaAsO_4$ (ICSD #415338) and $AlAsO_4$ (ICSD #201774) for different samples. The many-body amplitude reduction factor (S_0^2) was established as 0.95~1.05 by isolating and fitting the first-shell of As-O. The parameters such as interatomic distance (R), coordination number (CN), the difference in threshold energy (ΔE_0) and the Debye-Waller factor (σ^2) were first established with reasonable guesses and then fitted in R-space. The error in the overall fits was determined by the R-factor, called the goodness-of-fit parameter. It is defined as R-factor = $\Sigma(\chi_{data} - \chi_{fit})^2/\Sigma(\chi_{data})^2$. Good fits occur for R-factor < 0.05.

In the fitting process, multiple scattering (MS) paths were also considered. As-O-

O triangular (MS) paths were included in the As(III) and As(V) spectra fitting as reported in previous studies[9] (Table S4). Implementation of MS did not increase the number of variations in the fit because the MS path degeneracies were fixed to their theoretical values, and the Debye-Waller parameters, as well as mean half path lengths were related to the respective value of the As-O single scattering (SS) path. The degeneracy numbers of MS for As(III) and As(V) were fixed at the expected values of 12 and 6, respectively. The mean half path length of MS path for As(III) and As(V) was defined as $(1+\sin 50^{o})\times R_{As\text{-}O}$ and $(1+\frac{\sqrt{6}}{3})\times R_{As\text{-}O}$, respectively.[9] The Debye-Waller parameter of the MS path was $\sigma^2_{As\text{-}O}$.[9]

Table S4. Structure parameters derived from As K-edge EXAFS analysis.

Samples	Path	CN[a]	R (Å)[b]	σ^2 (Å^2)[c]	ΔE_0 (eV)[d]	R-factor[e]
As(III)-$La(OH)_3$	As-O	3[f]	1.79(2)	0.004(3)	5.1	0.017
	As-O-O	6[f]	3.16[f]	0.004(3)		
	As-La	1.9(13)	3.27(6)	0.009(8)		
As(III)-rGO/LOb	As-O	2.55[g]	1.79(1)	0.003(2)	3.4	0.018
	As-O-O	5.1[g]	3.16[f]	0.003(2)		
	As-La	1.6[g]	3.28(2)	0.009(6)		
	As-O	0.6[h]	1.69(1)	0.001(1)		
	As-O-O	1.8[h]	3.07[f]	0.001(1)		
	As-La	0.15[h]	3.35(1)	0.009(8)		
As(V)-$La(OH)_3$	As-O	4[f]	1.69(1)	0.002(1)	6.2	0.010
	As-O-O	12[f]	3.07[f]	0.002(1)		
	As-La	1.0(3)	3.35(3)	0.005(2)		
	As-La	2.9(13)	3.86(5)	0.011(8)		
As(V)-rGO/LOb	As-O	4[f]	1.69(1)	0.002(1)	6.6	0.011
	As-O-O	12[f]	3.07[f]	0.002(1)		
	As-La	1.0(4)	3.34(4)	0.006(3)		
	As-La	3.5(20)	3.86(5)	0.013(9)		

[a] Coordination number. [b] Interatomic distance. [c] Debye-Waller factor. [d] Threshold energy shift. [e] Goodness-of-fit parameter: R-factor = $\Sigma(\chi_{data} - \chi_{fit})^2/\Sigma(\chi_{data})^2$. [f] Fixed parameters. [g] Values were obtained by multiplying the fitting parameter for As(III) on $La(OH)_3$ with 85%. [h] Values were obtained by multiplying the fitting parameter for As(V) on $La(OH)_3$ with 15%. Parentheses: the estimated parameter uncertainties are listed in parentheses, representing the errors in the last digit; values without reported errors are fixed during fitting.

References:

[1] a) K. Zhang, L. L. Zhang, X. S. Zhao, J. S. Wu, *Chem Mater* **2010**, *22*, 1392-1401; b) S. B. Liu, T. H. Zeng, M. Hofmann, E. Burcombe, J. Wei, R. R. Jiang, J. Kong, Y. Chen, *Acs Nano* **2011**, *5*, 6971-6980; c) S. Park, J. An, J. R. Potts, A. Velamakanni, S. Murali, R. S. Ruoff, *Carbon* **2011**, *49*, 3019-3023.

[2] a) A. C. Ferrari, *Solid State Commun* **2007**, *143*, 47-57; b) I. K. Moon, J. Lee, R. S. Ruoff, H. Lee, *Nat Commun* **2010**, *1*; c) L. H. Tang, Y. Wang, Y. M. Li, H. B. Feng, J. Lu, J. H. Li, *Adv Funct Mater* **2009**, *19*, 2782-2789.

[3] a) D. L. Hoang, A. Dittmar, M. Schneider, A. Trunschke, H. Lieske, K. W. Brzezinka, K. Witke, *Thermochim Acta* **2003**, *400*, 153-163; b) L. M. Cornaglia, J. Munera, S. Irusta, E. A. Lombardo, *Appl Catal a-Gen* **2004**, *263*, 91-101.

[4] a) H.-J. Shin, K. K. Kim, A. Benayad, S.-M. Yoon, H. K. Park, I.-S. Jung, M. H. Jin, H.-K. Jeong, J. M. Kim, J.-Y. Choi, Y. H. Lee, *Adv Funct Mater* **2009**, *19*, 1987-1992; b) J. A. Brandes, G. D. Cody, D. Rumble, P. Haberstroh, S. Wirick, Y. Gelinas, *Carbon* **2008**, *46*, 1424-1434; c) H.-K. Jeong, H.-J. Noh, J.-Y. Kim, M. Jin, C. Park, Y. Lee, *EPL (Europhysics Letters)* **2008**, *82*, 67004; d) M. F. Sunding, K. Hadidi, S. Diplas, O. M. Løvvik, T. E. Norby, A. E. Gunnæs, *J Electron Spectrosc* **2011**, *184*, 399-409; e) W. Xie, L.-T. Weng, C.-K. Chan, K. L. Yeung, C.-M. Chan, *Phys Chem Chem Phys* **2018**, *20*, 6431-6439.

[5] a) M. Newville, *J. Synchrotron Radiat.* **2001**, *8*, 322-324; b) B. Ravel, M. Newville, *J. Synchrotron Radiat.* **2005**, *12*, 537-541.

[6] a) J. Du, J. Cui, C. Jing, *Chem. Commun.* **2014**, *50*, 347-349; b) C. Y. Jing, J. L.

Cui, Y. Y. Huang, A. G. Li, *ACS Appl. Mater. Interfaces* **2012**, *4*, 714-720.

[7] M. Newville, P. Livins, Y. Yacoby, J. J. Rehr, E. A. Stern, *Phys Rev B* **1993**, *47*, 14126-14131.

[8] J. M. Deleon, J. J. Rehr, S. I. Zabinsky, R. C. Albers, *Phys Rev B* **1991**, *44*, 4146-4156.

[9] a) C. Mikutta, R. Kretzschmar, *Environ. Sci. Technol.* **2011**, *45*, 9550-9557; b) Y. H. Wang, G. Morin, G. Ona-Nguema, N. Menguy, F. Juillot, E. Aubry, F. Guyot, G. Calas, G. E. Brown, *Geochimica Et Cosmochimica Acta* **2008**, *72*, 2573-2586.
